# Cementless Compared to Cemented Total Knee Arthroplasty is Associated With More Revisions Within 1 Year of Index Surgery

**DOI:** 10.1016/j.artd.2023.101122

**Published:** 2023-05-10

**Authors:** Daniel Chiou, Alan K. Li, Alexander Upfill-Brown, Armin Arshi, Peter Hsiue, Kevin Chen, Alexandra Stavrakis, Christos Demetris Photopoulos

**Affiliations:** a Department of Orthopaedic Surgery, University of California, Los Angeles, CA, USA; b Kerlan-Jobe Orthopaedic Institute, Los Angeles, CA, USA

**Keywords:** Total knee arthroplasty, Cementless, Cemented, Revision rates

## Abstract

**Background:**

Cementless total knee arthroplasties (TKAs) have gained renewed interest due to improved implant designs and lower rates of revision than its cemented counterparts. The purpose of this study was to compare revision rates between cemented vs cementless TKAs within 1 year of primary arthroplasty.

**Methods:**

This was a retrospective review from the PearlDiver Patient Record Database. International Classification of Diseases and Current Procedural Terminology codes were used to identify patients who had undergone cemented and cementless TKAs and subsequent surgical revisions. An unadjusted univariate analysis of patient demographics, Charlson Comorbidity Index score, and surgical revisions at 90 days and 1 year after TKA was performed using chi-squared testing. Multivariate logistic regression analyses were subsequently performed for 1-year surgical complications requiring revision.

**Results:**

Of 324,508 patients, 312,988 (96.45%) underwent cemented TKAs, and 11,520 (3.55%) underwent cementless TKAs. Patients undergoing cementless TKA tended to be younger than patients undergoing cemented TKA (63.67 ± 9.15 cementless vs 66.22 ± 8.85 cemented, *P* < .001). Univariate chi-squared testing showed that cementless patients were more likely to require 1-component femoral or tibial revision at 90 days and 1 year, irrigation and debridement at 90 days and 1 year, and arthroscopy with lysis of adhesions at 1 year only. Similar findings were observed for these 3 revision procedures at 1 year after correcting for age, gender, and Charlson Comorbidity Index score using multivariate logistic regression analysis as cementless TKA patients had higher odds ratios for each of the revisions.

**Conclusions:**

Small but significant differences were found in surgical revisions among cementless TKAs when compared to cemented TKAs within 1 year of the index procedure.

## Introduction

Total knee arthroplasty (TKA) is one of the most common and reliable surgical treatments for osteoarthritis in reducing pain, enhancing functional capacity, and improving quality of life and life expectancy [[1], [2], [3], [4], [5]]. In 30 years, the total annual number of TKA surgeries is set to increase by 401% [6]. However, alongside the growth in TKA is the increasing burden for revision TKA, estimated to pose more than a $13-billion burden on the US health-care system [[7], [8], [9]]. While most revision TKAs are to extend prosthesis longevity, outcomes of revision TKA have been shown to be poorer than their primary counterparts due in part to bone loss and soft-tissue insufficiency during revision surgeries [[10], [11], [12], [13], [14], [15], [16]].

Different types of TKA (cemented vs cementless) require revision surgeries for different reasons [[17], [18], [19]]. Historically, cementless TKAs were known for unstable fixation properties, poorly designed components, femoral component fractures, screw track osteolysis, patellar polyethylene wear, and failure of osteointegration leading to early subsidence [[20], [21], [22], [23], [24], [25], [26], [27], [28]]. Cemented TKAs, on the other hand, undergo gradual deformation and degradation over time and have weak resistance to shear forces leading to osteolytic activity at the cement-bone interface [18,29]. Still, other common causes of TKA failure leading to revisions are aseptic loosening, polyethylene wear, infection, instability, and periprosthetic fractures, thus requiring patients to undergo a revision surgery [8,14,[30], [31], [32], [33], [34]].

Recently, there has been a renewed interest in cementless TKA given the demographic shift from older to younger and more active patients that require long-term, stable fixations [30,35]. Cemented TKAs have higher failure rates among certain groups such as obese and younger patients [[36], [37], [38]]. The majority of the studies on cementless TKA were written prior to recent advancements such as porous surfaces, second-generation prosthesis designs and features, and new biomaterials that have demonstrated favorable functional results [17,[39], [40], [41], [42], [43], [44], [45], [46], [47], [48], [49]]. Due to the increased demand on younger, more active patients and the success of cementless TKA, the use of cementless TKA must be further analyzed. The purpose of this study was to investigate the revision rates between cementless and cemented TKA in the United States. We hypothesize that revision rates would be comparable between cementless and cemented TKA at 1-year follow-up.

## Material and methods

This study was a retrospective review of the MKnee data set of the PearlDiver Mariner Patient Claims Database (PearlDiver Technologies, Colorado Springs, CO). The MKnee data set is a commercial registry of 2 million insured patients who have undergone knee procedures from 2015 through 2019. Clinical diagnoses from patient records can be queried using International Classification of Diseases (ICD) and Current Procedural Terminology codes. All data are deidentified and considered exempt from institutional review board requirements.

ICD-10 diagnosis and procedure codes were utilized to identify patients and to create 2 sub-groups: those who had undergone cemented knee arthroplasty and those who had undergone uncemented knee arthroplasty. ICD-10 codes, which were implemented in the fourth quarter of 2015, include laterality and allow the differentiation between cemented and uncemented knee arthroplasty procedures that were utilized for this study. Patients were included in the analysis if they were at least 20 years of age at the time of index TKA (2015 q4 through 2019) and had a minimum of 1-year follow-up. Patients whose procedure codes did not specify laterality were excluded from the analysis.

Demographic data for each record were identified, including the patient’s age and gender. Presurgical medical comorbidities for records within each sub-group were also identified and defined when at least 1 instance of each comorbidity was diagnosed under any encounter in the patient record within 1 year prior to the index surgery. The Charlson Comorbidity Index (CCI) was also calculated for all records. The 2 subgroups were then queried to identify patients who had undergone a revision knee surgery at 90 days and 1 year from their index surgery, which were the primary outcomes of this study. Revision procedures that were queried included femoral/tibial single-component revision, femoral/tibial dual-component revision, knee explantation, irrigation and debridement (I&D), reduction status after dislocation, manipulation under anesthesia (MUA), arthroscopic lysis of adhesions, femur open reduction and internal fixation for periprosthetic fractures, and tibia open reduction and internal fixation for periprosthetic fractures.

Data analysis was performed using the PearlDiver R analysis package (PearlDiver Technologies, Colorado Springs, CO). Raw/unadjusted univariate analysis of demographic, comorbidity, and revision data was performed using chi-squared testing or Welch's *t*-testing where appropriate. A logistic multivariate regression analysis was performed at 90 days and 1 year from surgery to identify odds radios of postoperative revision procedures for uncemented procedures relative to cemented procedures. Demographic and presurgical comorbidities were included in the multivariate analysis as controls if there was a significant difference among cohorts seen in the univariate analysis. The multivariate analysis results were reported as odds ratios (OR) with 95% confidence intervals (CI). The initial model included all variables significantly associated with the outcomes of interest. A backwards selection using a metric of model fit was performed, which entailed removing any covariate not associated with mode outcome enough to improve the model fit. To decrease the risk of type I error, a Bonferroni correction was performed, and a resultant *P* value of <0.0028 was utilized to determine statistical significance.

## Results

### Patient demographics and comorbidities

From 2015 to 2019, 324,508 patients underwent cementless or cemented TKA and were included in the study. Of the total, 312,988 (96.45%) underwent cemented TKAs, and 11,520 (3.55%) underwent cementless TKAs. Females made up a smaller percentage of the cementless TKA group vs the cemented TKA group (57.67% uncemented TKA vs 63.48% cemented TKA, *P* < .001). Patients undergoing cementless TKA tended to be younger than patients undergoing cemented TKA (64.02 ± 9.2 cementless TKA vs 66.4 ± 8.9 cemented TKA, *P* < .001) and had less comorbidities as determined by the CCI (2.10 ± 2.3 cementless TKA vs 2.18 ± 2.4 cemented, *P* < .001) (Table 1).

**Table 1:** Patient demographics.

Demographic/comorbidity	Cemented (N = 312,988)		Cementless (N = 11,520)		
	n	%	n	%	*P* value
Age <40	987	0.32	62	0.54	<.001
Age 40-49	10,463	3.34	682	5.92	<.001
Age 50-59	59,079	18.88	2908	25.24	<.001
Age 60-69	116,569	37.24	4375	37.98	.112
Age ≥70	113,167	36.16	3082	26.75	<.001
Gender: M	114,313	36.52	4876	42.33	<.001
Gender: F	198,675	63.48	6644	57.67	
CCI = 0	79,524	25.41	2935	25.48	.875
CCI = 1	72,757	23.25	2883	25.03	<.001
CCI = 2	58,388	18.66	2101	18.24	.264
CCI = 3	37,042	11.83	1389	12.06	.477
CCI = 4	24,217	7.74	829	7.20	.034
CCI ≥ 5	41,060	13.12	1383	12.01	<.001

## Specific patient comorbidities

Regarding cardiovascular-related comorbidities, cementless TKA patients were less likely to have hypertension (56.90% cementless TKA vs 58.56 cemented TKA, *P* < .001), coagulopathy (2.24% cementless TKA vs 2.91 cemented TKA, *P* < .001), and coronary artery disease (11.94% cementless TKA vs 12.84% cemented TKA, *P* = .005). Regarding pulmonary comorbidities, cementless TKA patients were more likely to have asthma (8.33% cementless TKA vs 7.51% cemented TKA, *P* = .001) and chronic obstructive pulmonary disease (15.89% cementless TKA vs 15.09% cemented TKA, *P* = .01). Regarding metabolic comorbidities, cementless TKA patients were more likely to have obesity (23.45% cementless TKA vs 20.86% cemented TKA, *P* < .001) and liver disease (4.27% cementless TKA vs 3.74% cemented TKA, *P* = .003) but less likely to have hypothyroidism (13.99% cementless TKA vs 15.50% cemented TKA, *P* < .001), renal disease (5.78% cementless TKA vs 6.79% cemented TKA, *P* < .001), and history of cancer (5.16% cementless TKA vs 6.03% cemented TKA, *P* < .001). Cementless patients were more likely to display tobacco use (17.35% cementless TKA vs 8.89 cemented TKA, *P* < .001), alcohol abuse (1.61% cementless TKA vs 1.34 cemented TKA, *P* = .013), and drug abuse (2.51% cementless TKA vs 2.05 cemented TKA, *P* < .001). Cementless TKA patients were also more likely to have depression (14.96% cementless vs 14.01% cemented TKA patients, *P* = .004). No other significant differences in rates of comorbidities were observed between the 2 patient groups (Table 2).

**Table 2:** Specific Patient Comorbidities.

Patient comorbidities	Cemented TKA (N = 312,988)		Cementless TKA (N = 11,520)		*P* value
	n	%	n	%	
Cardiovascular					
Hypertension	183,297	58.56	6555	56.90	<.001
Coronary artery disease	40,181	12.84	1375	11.94	.005
Congestive heart failure	4132	1.32	128	1.11	.058
Coagulopathy	9107	2.91	43	2.24	<.001
Pulmonary					
Asthma	23,504	7.51	960	8.33	.001
Chronic obstructive pulmonary disease	46,972	15.01	1830	15.89	.010
Metabolic					
Diabetes	74,361	23.76	2776	24.10	.408
Obesity	65,275	20.86	2701	23.45	<.001
Liver disease	11,703	3.74	492	4.27	.003
Hypothyroidism	48,519	15.50	1612	13.99	<.001
Renal disease	21,253	6.79	666	5.78	<.001
History of cancer	18,870	6.03	594	5.16	<.001
Other					
Tobacco use	27,838	8.89	1999	17.35	<.001
Alcohol abuse	4190	1.34	186	1.61	.013
Drug abuse	6410	2.05	289	2.51	.001
Depression	43,845	14.01	1723	14.96	.004

Significance was defined as *P* < .05.

### Complications requiring surgical revisions

Cementless TKA patients were more likely than cemented TKA patients to require 1-component femoral or tibial revision at both 90 days (0.39% vs 0.21%, *P* < .001) and 1 year (0.87% vs 0.45%, *P* < .001). Cementless TKA patients were more likely than cemented TKA patients to require explantation at both 90 days (0.10% vs 0.05%, *P* = .012) and 1 year (0.32% vs 0.17%, *P* < .001). Cementless TKA patients were also more likely than cemented patients to require I&D at both 90 days (0.45% vs 0.25%, *P* < .001) and 1 year (2.47% vs 2.40%, *P* < .001). Cementless TKA patients were more likely than cemented patients to require arthroscopy with lysis of adhesions (LOA) at both 90 days (0.10% vs 0.03%, *P* < .001) and 1 year (0.37% vs 0.19%, *P* < .001). At 1 year, cementless TKA patients were also more likely than cemented TKA patients to require both component femoral and tibial revision (0.43% vs 0.30%, *P* < .001) and MUA (3.53% vs 3.17%, *P* = .034). No other significant differences in surgical revision rates were observed at 90 days or 1 year (Table 3, Table 4).

**Table 3:** Raw/unadjusted univariate analysis of revision data at 90 days.

Revision procedure	Cemented TKA (N = 312,988)		Cementless TKA (N = 11,520)		*P* value
	n	%	n	%	
Femoral/tibial revision, 1 component	648	0.21	45	0.39	<.001
Femoral/tibial revision, both components	169	0.05	<11	N/A	N/A
Explantation	147	0.05	12	0.10	.012
Irrigation and debridement	787	0.25	52	0.45	<.001
Reduction status, after dislocation	17	0.01	<11	N/A	N/A
Manipulation under anesthesia	7505	2.40	285	2.47	.622
Arthroscopy with lysis of adhesions	103	0.03	12	0.10	<.001
ORIF, femur PPFx	26	0.01	0	0.00	N/A
ORIF, tibia PPFx	11	N/A	0	0.00	N/A

ORIF, open reduction and internal fixation; PPFx, periprosthetic fracture.

Significance was defined as *P* < .05.

**Table 4:** Raw/unadjusted univariate analysis of revision data at 1 year.

Revision procedure	Cemented TKA (N = 312,988)		Cementless TKA (N = 11,520)		*P* value
	n	%	n	%	
Femoral/tibial revision, 1 component	1402	0.45	100	0.87	<.001
Femoral/tibial revision, both components	949	0.30	49	0.43	.025
Explantation	532	0.17	37	0.32	<.001
Irrigation and debridement	1168	0.37	70	0.61	<.001
Reduction status, after dislocation	42	0.01	<11	N/A	N/A
Manipulation under anesthesia	9936	3.17	407	3.53	.034
Arthroscopy with lysis of adhesions	591	0.19	43	0.37	<.001
ORIF, femur PPFx	48	0.02	<11	N/A	N/A
ORIF, tibia PPFx	17	0.01	0	0.00	N/A

ORIF, open reduction and internal fixation; PPFx, periprosthetic fracture.

Significance was defined as *P* < .05.

After correcting for statistically significant demographics, including age, gender, and CCI, multivariate logistic regression revealed that cementless TKA patients were more likely than cemented TKA patients to require 1-component femoral or tibial revision at 90 days (OR = 1.80, 95% CI = 1.31-2.41, *P* < .001) and 1 year (OR = 1.00, CI = 1.45-2.18, *P* < .001). Cementless TKA patients were also more likely to require I&D at 90 days (OR = 1.67, CI = 1.24-2.19, *P* < .001) and 1 year (OR = 1.51, CI = 1.19-1.93, *P* < .001). Compared to cemented TKA patients, cementless TKA patients were more likely to require arthroscopy with LOA at 90 days (OR = 2.76, CI = 1.43-4.83, *P* < .001) and 1 year (OR = 1.73, CI = 1.25-2.32, *P* < .001). Lastly, cementless TKA patients were more likely to undergo explantation at 1 year (OR = 1.68, CI = 1.20-2.35, *P* = .002). There were no other significant differences in ORs of surgical revisions (Table 5).

**Table 5:** Multivariate logistic regression of revision procedures at 90 days and 1 year.

Revision procedure	90 d		1 y	
	Cementless odds ratio (95% confidence interval)	*P*-value	Cementless odds ratio (95% confidence Interval)	*P* value
Femoral/tibial revision, 1 component	1.80 (1.31-2.41)	<.001	1.79 (1.45-2.18)	<.001
Femoral/tibial revision, both components	1.47 (0.70-2.72)	.2602	1.31 (0.97-1.73)	.067
Explantation	1.96 (1.03-3.38)	.026	1.68 (1.20-2.35)	.002
Irrigation and debridement	1.67 (1.24-2.19)	<.001	1.51 (1.19-1.93)	.001
Dislocation	1.67 (0.09-8.18)	.618	1.62 (0.26-5.35)	.507
Manipulation under anesthesia	0.91 (0.80-1.02)	.119	0.98 (0.89-1.09)	.763
Arthroscopy with lysis of adhesions	2.76 (1.43-4.83)	<.001	1.73 (1.25-2.32)	<.001
ORIF, femur PPFx	N/A	N/A	2.55 (0.77-6.27)	.073
ORIF, tibia PPFx	N/A	N/A	N/A	N/A

ORIF, open reduction and internal fixation; PPFx, periprosthetic fracture.

Significance was defined as *P* < .0028 after Bonferroni correction.

## Discussion

Our study found that at both the 90-day and 1-year mark, there was a small but significant incidence of I&D among uncemented arthroplasties. In addition, at the 1-year mark, there were similar small but significant incidences of 1-component revision and arthroscopy with LOA among uncemented arthroplasties when compared to the cemented counterparts.

Cemented TKAs have increased failure rates among obese patients [36,50]. There are also higher failure rates among younger patients, with rates of revision almost 4.7× greater for patients younger than 50 years [51,52]. Theoretically, cementless TKAs utilize biologically osseointegrated surfaces to avoid third body wear and potential fatigue and failure from repetitive loading [24]. Thus, aseptic loosening is one of the most common indications for revision TKA [14]. Multiple studies have demonstrated no significant differences among revision rates between cemented and cementless fixation methods. Miller et al. showed that at a minimum of 2-year follow-up, cemented and cementless TKAs had similar rates of failure leading to revision, with a higher incidence of aseptic loosening in the cement group (0.5% vs 2.5%) that was not significant [19]. The meta-analysis by Prasad et al. showed no significance difference in revision rates between cemented and cementless TKAs up to 16.6 years [24]. Similarly, Fricka et al. reported equivalent survivorship (revision for any reason as the endpoint) between cementless TKA and cemented TKA at the 2-year postoperative period [40]. However, both Fricka et al. and Choy et al. noted there were more radiolucent lines (RLLs) seen in cementless fixation than in cemented TKAs but did not demonstrate a significance [24,40,53]. RLLs are conventionally considered as results of osteolysis due to polyethylene or aseptic loosening. But RLLs can also be observed early after TKA secondary to the surgical technique with insufficient bone preparation, mispositioning of components, or inadequate cement penetration into sclerotic bone [54]. When asymptomatic, RLLs can be followed up closely with radiographs. When symptomatic, RLLs can be an indication for revision. Choy et al. discussed that in their posterior cruciate ligament sacrificing mobile-bearing low contact stress TKA, RLLs were observed only in the tibial component of both the cemented (8%) and cementless groups (13%), with no clinical significance in loosening or osteolysis at the 8- to 11-year postoperative period [53]. In contrast, our data demonstrated a significant difference in 1-component revision at the 1-year mark between cemented and cementless TKAs. Although the actual percentages are small, the clinical significance lies in the individual patients who still require revision surgeries, as they pose a huge financial, emotional burden to both patients and providers.

The second most common cause of revision TKAs is periprosthetic joint infection (PJI), which has an incidence of 1.5%-2% of all TKA procedures [[55], [56], [57]]. One of the forms of treatment include I&D. However, similar to aseptic loosening as a cause for revision, studies have shown equivocal results between cemented and cementless TKAs, with PJI as a cause for revision. Liu et al. performed a meta-analysis including 16 studies which showed that PJI as a cause of revision was 1.1% for cementless and 1.3% for cemented TKA with no significance [58]. Wang et al. also showed that there was no significant difference between these 2 groups with respect to infection [59]. Fricka et al. similarly showed that at the 2-year postoperative mark, there was no difference between cemented and cementless TKAs with respect to infection [40]. In contrast, there was a significant difference at both the 90-day and 1-year marks for patients undergoing 1 I&D.

Another significant complication following TKA is stiffness as a result of arthrofibrosis, which leads to pain, stiffness, functional disability, and patient morbidity [60,61]. MUA is the first-line consideration in patients who do not respond to physical, while arthroscopy for LOA is saved for those refractory to nonoperative management [61]. The difference we found between cemented and cementless fixations was the need for MUA and arthroscopy for LOA at the 1-year mark. Fricka et al. showed that functional scores were higher in the cemented group at the 2-year mark, but not in the cementless group [40]. Prasad et al., however, found that there was no significant difference in functional scores between the 2 groups (*P* > .05), which was similar to the conclusion of Kim et al [24,62]. Our data showed an increased incidence of arthroscopy for LOA at the 1-year mark for cementless TKAs. Interestingly, our study demonstrated an increased incidence and significant difference in 1-component revision, I&D, and arthroscopy compared to other studies. One component of this difference could be time of follow-up, as all of our follow-ups occurred at the 1-year period. There may be increased failure rates due to cementation failure, leading to the possible nonsignificant differences in other [30,63]. As more data are accumulated for longer periods of time, we predict there may be nonsignificance between cementless and cemented TKAs. Another factor for the difference is the sample size: The study by Liu et al. included about 5000 patients, Prasad et al. about 700, Miller et al. about 400 patients, and Fricka et al. 93 patients [19,24,40,58]. These are all a smaller sample size than the more than 300,000 patients in this study, which can contribute to the difference we uncovered.

There are a few limitations to our study, which contributed the differences in data between ours and other studies. Because the data source was a large administrative database, there is limited information regarding specific individual patient and surgeon. For example, certain cementless implant models have higher rates of failure, and since PearlDiver does not delineate this information, our study could be confounded by this information [64,65]. In addition, because PearlDiver contains private insurance claims (mostly from United Healthcare), the southern American states, where private insurance is utilized commonly, may be demographically overrepresented in this study [66]. This would limit and potentially confound the results of this study. Although a brief review of recent ICD-10 audits did not demonstrate any coding errors specific to the procedures involved in this project, there is potential for loss of patient inclusion in this study [67]. Certain procedures, such as polyethylene exchange and patella resurfacing, were also not included in the study, which could have confounded our results for “revision surgery.” Finally, our study was limited to the 1-year postoperative period. Many of these complications extend beyond this short time period, and it may be possible that the data lose significance with extended postoperative follow-up.

## Conclusions

Compared to cemented TKA, cementless TKA is associated with a small but increased rate of I&D, 1-component revision, and arthroscopy with LOA among uncemented arthroplasties when compared to the cemented counterparts. Although longer follow-up and individualized patient data are necessary to delineate these differences, this study is significant in identifying patterns of reoperations in cementless TKAs up to 1 year after the index surgery.

## Conflicts of interest

Dr. A. Stavrakis is paid consultant for DePuy and is a board member of American Association of Hip and Knee Surgeons. All other authors declare no potential conflicts of interest.

For full disclosure statements refer to https://doi.org/10.1016/j.artd.2023.101122.

## Appendix A Supplementary Data

Conflict of Interest Statement for Arshi

Conflict of Interest Statement for Chen

Conflict of Interest Statement for Hsiue

Conflict of Interest Statement for Chiou

Conflict of Interest Statement for Photopoulos

Conflict of Interest Statement for Stavrakis

Conflict of Interest Statement for Upfill-Brown

Conflict of Interest Statement for Li
